# Molecular Pathophysiology of Chronic Thromboembolic Pulmonary Hypertension: A Clinical Update from a Basic Research Perspective

**DOI:** 10.3390/arm92060044

**Published:** 2024-11-27

**Authors:** Leslie Marisol Gonzalez-Hermosillo, Guillermo Cueto-Robledo, Dulce Iliana Navarro-Vergara, Maria Berenice Torres-Rojas, Marisol García-Cesar, Oscar Pérez-Méndez, Galileo Escobedo

**Affiliations:** 1 Laboratory of Immunometabolism, Research Division, General Hospital of Mexico “Dr. Eduardo Liceaga”, Mexico City 06720, Mexico; glez.hermosillo.m@gmail.com; 2 Cardiorespiratory Emergency Department, Pulmonary Hypertension Clinic, General Hospital of Mexico “Dr. Eduardo Liceaga”, Mexico City 06720, Mexico; gmocue3@hotmail.com (G.C.-R.); dulceiliana@hotmail.com (D.I.N.-V.); beretorres.m.d@hotmail.com (M.B.T.-R.); shak_mary@hotmail.com (M.G.-C.); 3 Doctorate Program in Biomedical Sciences, Postgraduate Unit, National Autonomous University of Mexico, Mexico City 04510, Mexico; 4 Tecnológico de Monterrey, School of Engineering and Sciences, Mexico City 14380, Mexico; opmendez@yahoo.com; 5 Departamento de Biología Molecular, Instituto Nacional de Cardiología Ignacio Chávez, Mexico City 14080, Mexico

**Keywords:** chronic thromboembolic pulmonary hypertension, acute pulmonary embolism, fibrotic thrombus formation, pulmonary vascular remodeling, pulmonary hypertension, gene expression

## Abstract

**Highlights:**

**What are the main findings?**

- We explain the mechanisms involved in CTEPH development, including fibrotic thrombus formation, pulmonary vascular remodeling, and abnormal angiogenesis, which lead to elevated pulmonary vascular resistance and right heart failure.
- Improved diagnostic tools, biomarker identification, and therapeutic strategies are still needed to enhance early detection and management of CTEPH, ultimately aiming to reduce diagnostic delay and improve patient outcomes.

**What is the implication of the main finding?**

- A better understanding of CTEPH progression, including both proximal and distal obstruction of pulmonary arteries associated with the remodeling of pulmonary arteries.
- This narrative review summarizes the risk factors predicting CTEPH, including thrombotic history, hemostatic disorders, and certain medical conditions that help identify CTEPH progression and detection.

**Abstract:**

Chronic thromboembolic pulmonary hypertension (CTEPH) is a rare but severe condition characterized by persistent obstruction and vascular remodeling in the pulmonary arteries following an acute pulmonary embolism (APE). Although APE is a significant risk factor, up to 25% of CTEPH cases occur without a history of APE or deep vein thrombosis, complicating the understanding of its pathogenesis. Herein, we carried out a narrative review discussing the mechanisms involved in CTEPH development, including fibrotic thrombus formation, pulmonary vascular remodeling, and abnormal angiogenesis, leading to elevated pulmonary vascular resistance and right heart failure. We also outlined how the disease’s pathophysiology reveals both proximal and distal pulmonary artery obstruction, contributing to the development of pulmonary hypertension. We depicted the risk factors predicting CTEPH, including thrombotic history, hemostatic disorders, and certain medical conditions. We finally looked at the molecular mechanisms behind the role of endothelial dysfunction, gene expression alterations, and inflammatory processes in CTEPH progression and detection. Despite these insights, there is still a need for improved diagnostic tools, biomarkers, and therapeutic strategies to enhance early detection and management of CTEPH, ultimately aiming to reduce diagnostic delay and improve patient outcomes.

## 1. Introduction

Acute pulmonary embolism (APE) occurs when a thrombus, often originating in the deep veins, blocks one or more lung arteries [1]. Patients who survive an APE episode are at increased risk of developing post-pulmonary embolism (PE) syndrome [2], a medical condition encompassing several complications of PE, such as dyspnea, chest pain, and, more importantly, chronic thromboembolic pulmonary hypertension (CTEPH) [3,4,5].

CTEPH is a rare and potentially life-threatening disease characterized by elevated blood pressure in the pulmonary arteries that involves pulmonary vascular remodeling, chronic thrombotic mechanical blockage, and aberrant clearance of APE [6]. Specifically, CTEPH leads to microvascular remodeling consisting of fibrotic thrombi formation that can partially or entirely obstruct the pulmonary arteries, resulting in pulmonary vascular resistance (PVR) elevation, pulmonary hypertension (PH), and right heart failure [7,8]. The way to diagnose CTEPH is through right heart catheterization [9].

Despite accumulating evidence supports the causal role of APE in CTEPH occurrence, up to 25% of patients with CTEPH do not have a history of APE or deep vein thrombosis (DVT), suggesting the pathogenesis of CTEPH remains to be fully elucidated [10]. In this sense, there is not only evidence demonstrating a severe underestimation of CTEPH cases, but also inadequate medical knowledge of the illness combined with ineffective diagnosis that delays CTEPH treatment as much as 12 months or even longer [4,11,12,13]. Additionally, growing information shows that pulmonary arterial pressure (PAP) does not always correlate with the angiographic pulmonary vascular bed obstruction level [14]. Moreover, recent clinical studies reveal that PH can increase without recurrent venous thromboembolism (VTE). At the same time, current evidence confirms that pulmonary vascular resistance (PVR) is significantly higher in CTEPH patients than in APE subjects with similar vascular bed obstruction percentages [15,16]. For instance, several reports show CTEPH patients with around 35% pulmonary vascular obstruction and elevated PAP and PVR, while APE individuals with about 65% blockage can exhibit regular PAP and PVR values. This controversial clinical evidence suggests additional factors contributing to increased PH, PAP, and PVR besides pulmonary vascular blockage, as is the case of intimal and medial pulmonary artery hypertrophy occurring during pulmonary vascular remodeling [16].

The potential role of pulmonary vascular remodeling and gene expression profiles in the vascular endothelium illustrates the multiple cellular and molecular avenues still unexplored in CTEPH that may help better understand its etiology and medical therapeutic targets [17]. Herein, we will summarize the epidemiology, risk factors, and clinical presentation of CTEPH, focusing on describing the main pathophysiological mechanisms at the tissue, cellular, and molecular levels involved in CTEPH occurrence and progression (Table 1).

## 2. Epidemiology

CTEPH is a relatively rare disease compared to other forms of PH. The global prevalence of CTEPH is still uncertain, but it is estimated to be approximately 3.2 cases per million [40]. The cumulative incidence of CTEPH accounts for around 0.1–9.1% of patients who survived an APE episode within two years before [40,41]. The prevalence of CTEPH varies by region and healthcare resources, being higher in areas with an increased prevalence of VTE, such as Europe and Canada [4]. However, the notion that CTEPH is more common in developed countries can be attributed to developed nations’ better diagnosis schemes and access to healthcare than developing countries.

CTEPH can affect individuals of any age, but diagnosis occurs more often in people between 40 and 80 [42]. CTEPH can also affect both men and women, with a slight predominance in females [43].

## 3. Risk Factors and Clinical Presentation

**Figure 1:**
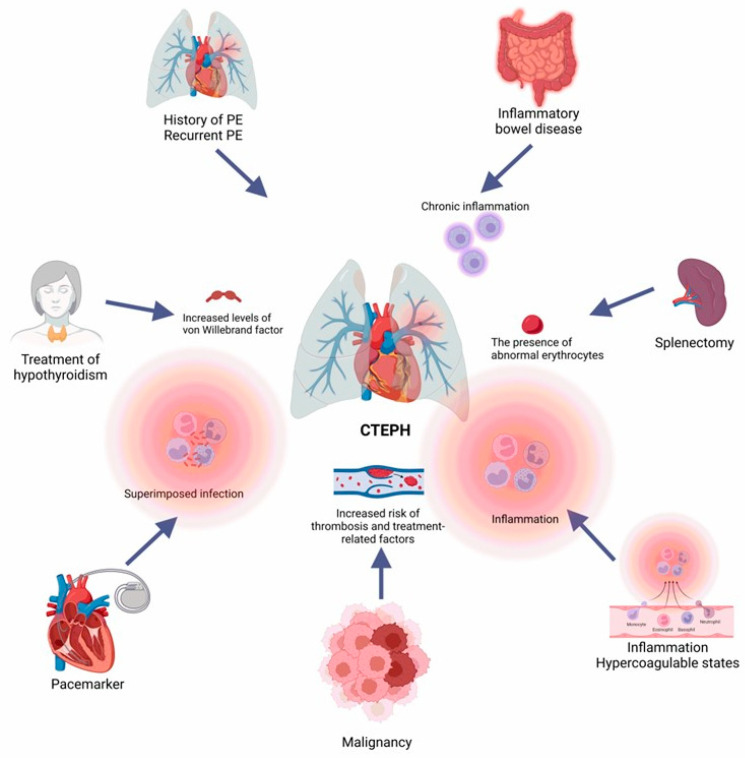
Primary risk factors for the development of chronic thromboembolic pulmonary hypertension. Previous episodes of pulmonary embolism, coagulation disorders, splenectomy, chronic infections, hypothyroidism treatment, cancer, and prolonged inflammatory states contribute to CTEPH development. These factors lead to persistent vascular obstruction and remodeling and increased pulmonary resistance, which are crucial features for CTEPH progression and aggravation. PE: pulmonary embolism; CTEPH: chronic thromboembolic pulmonary hypertension.

We categorize risk factors predisposing patients to CTEPH into three main groups. First, the most significant risk factor for CTEPH is having one or more APE episodes. APE-related risk factors include the thrombus’ intrinsic characteristics and persistent pulmonary vascular occlusion, which occur in almost four in ten APE patients [44,45,46]. Second, hemostatic risk factors contribute to CTEPH, especially coagulation cascade alterations and antibody response against factor VIII, which increase the risk of blood clot formation [47]. Third, numerous medical conditions appear to increase the chance of developing CTEPH, such as cancer, thrombophilia, genetic susceptibility, inflammatory bowel disease, autoimmune disorders, hypothyroidism, obesity, smoking, and long-term use of intravascular catheters [48]. Surgeries, especially orthopedic procedures and splenectomy, are associated with the development of blood clots and an increased risk of CTEPH [49]. A few studies have also suggested that long-term exposure to contraceptive pills or hormone replacement therapy may promote blood clot formation and lead to CTEPH (Figure 1).

Although we better understand the risk factors potentially contributing to CTEPH, our ability to predict which patients are at increased risk of developing this condition is still insufficient, especially in patients experiencing a first episode of APE. For this reason, multiple efforts currently focus on designing novel clinical tools that integrate the most representative medical, hemostatic, and biochemical variables for assessing the risk of developing CTEPH 6 to 24 months after being diagnosed with APE [50]. However, clinical scales should consider that around 25% of CTEPH patients do not have a known history of APE, which allows for us to see the enormous research area ahead in CTEPH etiology [51,52].

The clinical presentation of CTEPH may vary from patient to patient but includes numerous symptoms that we will outline further. Briefly, CTEPH patients may experience dyspnea or shortness of breath accompanied by chest pain, which often appears with physical activity [53]. Syncope or fainting episodes can occur in severe CTEPH cases due to a sudden decrease in cardiac output [54]; as the disease progresses, patients may also exhibit symptoms of right heart failure, such as jugular venous distention and hepatomegaly. Moreover, CTEPH patients may occasionally show hemoptysis or coughing up blood, fatigue, exercise intolerance, or peripheral edema, depending on the disease severity [55]. Another phenomenon often reported is that cardiologists can also incidentally detect CTEPH cases when performing medical tests to diagnose heart conditions, such as an electrocardiogram [54,56].

## 4. CTEPH Diagnosis

CTEPH diagnosis begins when a patient with a history of APE or unexplained pulmonary hypertension presents with progressive shortness of breath, fatigue, palpitations, or exercise intolerance and has been unresponsive to standard anticoagulation therapy for three months [57]. In this scenario, the first diagnostic tool is echocardiography, a noninvasive imaging technique that helps identify elevated pressures in the pulmonary artery and evidence of right ventricular strain [58]. Echocardiography can suggest pulmonary hypertension but cannot definitively diagnose CTEPH because it does not visualize thrombi within the pulmonary arteries. Then, physicians with training in cardiopulmonary hemodynamics can perform right heart catheterization (RHC) as a definitive procedure for diagnosing pulmonary hypertension [59]. RHC is an invasive test that directly measures pulmonary artery pressures, cardiac output, and vascular resistance. Thus, RHC provides a detailed hemodynamic profile crucial for diagnosing pulmonary hypertension and assessing its severity. The hemodynamic profile of CTEPH is characterized by pre-capillary pulmonary hypertension with a mean pulmonary arterial pressure (mPAP) > 20 mmHg, a pulmonary arterial wedge pressure (PAWP) ≤ 15 mmHg, and a pulmonary vascular resistance (PVR) > 2 Wood units [60]. Complementary imaging of pulmonary arteries by ventilation–perfusion (V/Q) scanning is critical to confirm CTEPH because it provides a noninvasive and highly sensitive method for detecting perfusion defects within the pulmonary vasculature [61]. In patients with CTEPH, the V/Q scan typically reveals mismatched perfusion defects, where lung regions receive adequate ventilation but have reduced blood flow due to vascular obstruction. V/Q scanning is preferred as an initial imaging test over computed tomography (CT) angiography in CTEPH due to its high sensitivity for detecting chronic perfusion defects. However, CT angiography is often used in the subsequent stages of the diagnostic process. Additionally, CT pulmonary angiography (CTPA) provides detailed anatomical images of the pulmonary arteries and is an essential imaging technique for directly visualizing thrombotic material [62]. In CTEPH patients, CTPA may show signs of chronic thromboembolic disease, such as organized clots, webs, bands, and stenotic areas within the pulmonary arteries [62]. CTPA is particularly useful for assessing the extent and distribution of thromboembolic obstructions and identifying patients who might benefit from surgical intervention. Lastly, CTEPH assessment includes laboratory tests and screening for conditions that may predispose patients to hypercoagulability, as these can increase the risk of recurrent thromboembolic events [63]. Testing for antiphospholipid antibodies, genetic clotting disorders, and other thrombophilias can help identify a patient’s risk factors and guide management strategies [64,65,66]. Combining clinical assessment, hemodynamic evaluation, advanced imaging, and laboratory testing allows for a precise diagnosis of CTEPH.

## 5. CTEPH Pathophysiology

**Figure 2:**
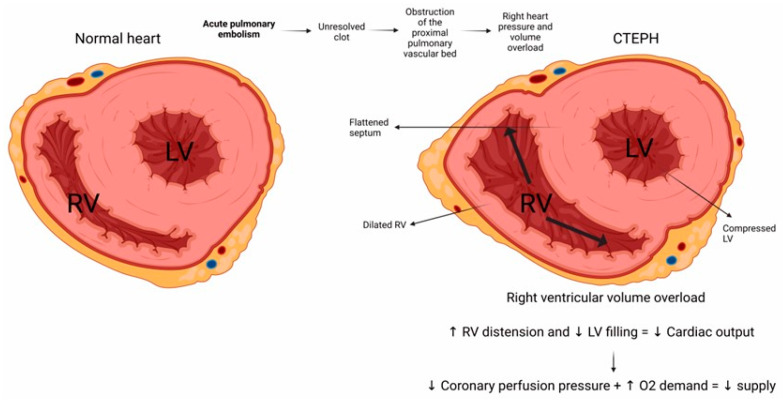
Cross-section representation showing heart–lung interactions in chronic thromboembolic pulmonary hypertension. In CTEPH, pulmonary vascular obstruction due to an unresolved clot increases pulmonary vascular resistance, resulting in right ventricular pressure overload. This overload causes RV hypertrophy and dilation, reduced cardiac output, and impaired ventriculoarterial coupling. The bidirectional interaction between the heart and lungs exacerbates right heart failure and contributes to the progressive deterioration of patients that conduces to less coronary perfusion pressure, increased oxygen demand, and finally, death. RV: right ventricular; LV: left ventricular; CTEPH: chronic thromboembolic pulmonary hypertension; O2: oxygen; ↑, increase; ↓, decrease.

CTEPH pathophysiology centrally involves the presence of persistent thrombi that become organized within large or middle-sized pulmonary arteries, leading to progressively increased lung hypertension [60]. After the pulmonary blood flow is partially or fully blocked, the pulmonary artery pressure arises, thus imposing strain on the heart’s right ventricle [67]. In this scenario, vascular remodeling, inflammation, and pulmonary vascular resistance interplay in a complex network that ultimately leads to right heart failure and death (Figure 2) [68].

**Figure 3:**
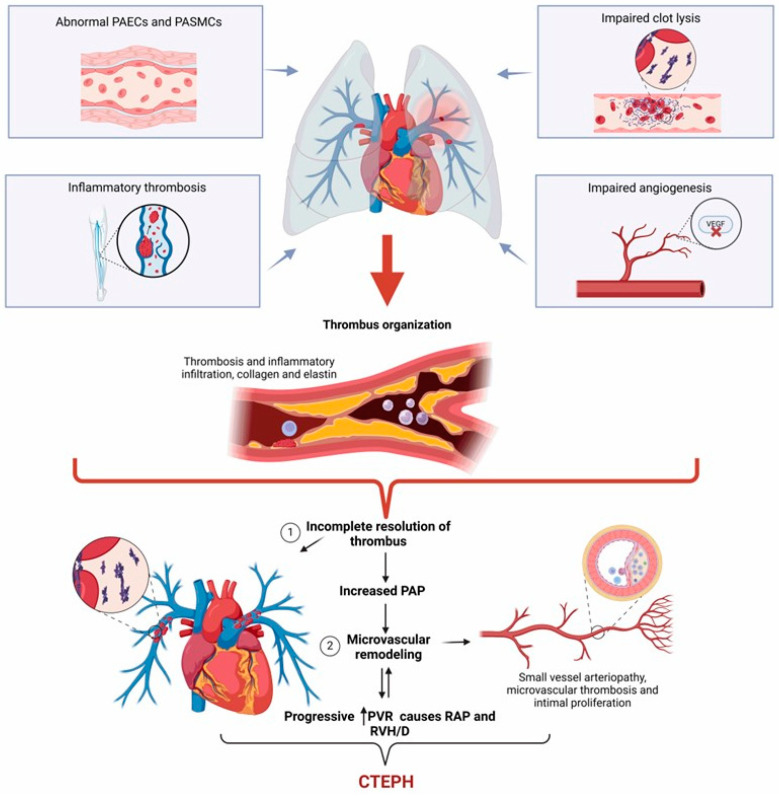
Pathophysiology of chronic thromboembolic pulmonary hypertension. Following acute pulmonary embolism, unresolved thrombus leads to persistent vascular obstruction. This phenomenon triggers a cascade of events, including increased hypercoagulability, abnormal clot lysis, impaired angiogenesis, and inflammatory thrombosis. Chronic inflammation and impaired fibrinolysis result in fibroblast and smooth muscle cell proliferation, inflammatory cell infiltration, and deposition of collagen and elastin, contributing to the formation of fibrotic, non-resolving thromboembolic material, also referred to as organized thrombi. These events also increase pulmonary artery pressure and vascular resistance, contributing to microvascular remodeling. The interplay of these factors results in right ventricular strain, a central feature of CTEPH pathogenesis. PASMCs: pulmonary artery smooth muscle cells; PAECs: pulmonary artery endothelial cells; PAP: pulmonary artery pressure; RVP: pulmonary vascular resistance; RAP: right atrial pressure; RVH: right ventricle hypertrophy; RHD: right ventricle dysfunction; CTEPH: chronic thromboembolic pulmonary hypertension.

CTEPH often develops after an APE episode, where most emboli dissolve after anticoagulant therapy [69]. However, in some cases, the emboli do not resolve and remain lodged in the pulmonary arteries, becoming chronic and organized with the ability to adhere to the walls of blood vessels rather than dissolving [64]. Thrombus organization occurs when fibrotic material gradually replaces the embolus to form bands or septa that can integrate into the vessel wall, which narrows both central and distal segments of the pulmonary arteries and increases vasoconstriction [64]. The obstructive effects of organized thrombi conduce to vascular remodeling, consisting of thickening of the muscle layer of the vessel walls, intimal hyperplasia and fibrosis, and plexiform lesion formation [70]. Inflammation also plays a role in CTEPH progression by recruiting immune cells into vessel lesions, where they produce cytokine and mediators such as tumor necrosis factor-alpha (TNF-alpha) or transforming growth factor-beta 1 (TGF-beta 1) [71]. TNF-alpha and TGF-beta 1 can induce endothelial cell apoptosis and artery vessel fibrosis, respectively, thus contributing to vascular remodeling and increased pulmonary vascular resistance [72,73]. As pulmonary vascular resistance rises, the right ventricle works harder to pump blood through the lungs, making the heart thicken the muscle wall, which leads to right ventricular dilation and, ultimately, right heart failure and death (Figure 3) [74,75].

We can distinguish CTEPH from APE according to the thromboembolic component features. The histological lesion of APE appears as a reddish new thrombus with a fibrin network primarily made up of red blood cells and platelets [76]. Conversely, thrombi found in CTEPH are yellowish and show a porous thrombus recanalization made of fibrous components such as collagen and elastin, inflammatory cells, and fibroblasts, often accompanied by calcifications [77]. An analysis of nine patients revealed the presence of foci resembling hemangiomatosis in lung samples of CTEPH patients. The study also documented notable venous remodeling, engagement of distal pulmonary arteries, and microvessel formation [8]. This evidence suggests that both pre-capillary and post-capillary pulmonary vasculature plays a crucial role in CTEPH development.

Patients with CTEPH exhibit two anatomical patterns of pulmonary vascular abnormalities leading to elevated PVR and vascular bed obstruction in pulmonary arteries. First, fibrotic clots can block the pulmonary arteries in the larger lobar-to-segmental or smaller subsegmental arteries [78]. Second, non-thrombotic obstructive remodeling involves narrowing pulmonary arterioles and capillaries, thus contributing to microvasculopathy [79]. Microvasculopathy in the unobstructed vascular areas reflects flow redirection from the obstructed territories, which shears stress from high flow and pressure. Then, high flow from the systemic collateral circulation originating in bronchial, intercostal, or coronary arteries redirects to significant vascular obstructions, connecting to pulmonary arteries and veins [69]. About one-third of patients may have elevated left ventricular filling pressures, which could be a factor in developing a post-capillary PH component [80]. Histopathologic evidence of medial hypertrophy, plexiform lesions, and intimal fibrosis indicates small-vessel PH [81].

## 6. Molecular Mechanisms in CTEPH

Most patients have a resolution of thrombi within three months following an acute episode of pulmonary embolism attributed to a multifaceted process of reorganization and recanalization [82]. Despite appropriate anticoagulant therapy that may prevent CTEPH, a causal link between these risk factors has not been proven [83]. The development of CTEPH is the result of a series of interrelated events that begin with the incomplete resolution and organization of the thrombus, followed by an anomaly in thrombus angiogenesis, concluding with the obstruction of large pulmonary arteries by fibrothrombotic lesions and the adaptive remodeling of pulmonary pre-capillary vessels [84]. A recent study shows that the plasminogen activator inhibitor-1 (PAI-1) contributes to impaired clot dissolution, a vital feature of the chronic nature of thromboembolism in CTEPH [20].

Unresolved fibrotic clots can cause intravascular blockage of the primary, lobar, segmental, and subsegmental pulmonary arteries, extending to the distal pulmonary arteries at the intra-acinar level. On vascular imaging, these histologic lesions appear as slits, webs, stenosis, or pouching. Some obstructive chronic thromboembolic lesions manifest distally as many secondary lumina and occluded arteries with recanalization, known as colander-like lesions [77]. CTEPH begins as an initial thromboembolic disease that does not resolve itself. A study demonstrated that fibrin from CTEPH patients was resistant to thrombolysis in vitro [81]. Together, inflammation associated with thrombosis leads to an increased prevalence of inflammatory diseases and inflammatory cell infiltration in the fibrothrombotic material obstructing the proximal pulmonary arteries. In parallel, the increased expression of interferon gamma-induced protein (IP)-10 promotes T cell adhesion to endothelial cells and inhibits bone marrow colony formation and angiogenesis [24]. Additionally, nuclear factor kappa B (NF-κB) upregulation induces endothelial cell dysfunction in CTEPH, contributing to increased expression of IL-8, IL-1 beta, CCL5, and MCP-1 [25,26]. Moreover, neutrophil extracellular traps contribute to the promotion of fibrous occlusions in chronic thrombosis together with an increased level of circulating inflammatory mediators, an increased level of factor VIII, and the exploration of epigenetic modification of the von Willebrand factor promoter behind platelet aggregation on the pulmonary endothelium.

Coagulation system abnormalities that lead to a hypercoagulability state can also contribute to chronic thrombosis [85]. Proteins C and S and antithrombin deficiency (classic hereditary risk factors for thrombosis) do not significantly differ between the CTEPH and control groups [86]. However, CTEPH is associated with increased plasma levels of factor VIII, lupus anticoagulant, and antiphospholipid antibodies [87,88]. The causes of thrombus non-resolution are still not wholly understood, just like the other mechanisms implicated in the pathophysiology of CTEPH. In control subjects, granulation tissue formation occurs after rapid fibrinolysis, as in the wound healing response. A cellular response then results in the recruitment of leukocytes, endothelial progenitor cells, and concurrent angiogenesis mediators [89,90,91]. Neutrophils infiltrate the resolving thrombus, supporting continued fibrinolysis and collagen degradation. Monocytes come immediately after neutrophils and differentiate into macrophages, performing a more significant role in promoting thrombus reorganization by secreting different chemokines, growth factors, and proteases [92].

Abnormalities in angiogenesis during thrombus resolution also contribute to the etiology of CTEPH [64]. Positive neovascularization regulators, such as vascular endothelial growth factor (VEGF) and fibroblast growth factor (FGF), increase and result in endothelial activation during normal angiogenesis [93]. We can confirm this phenomenon by experimental disruption of endothelial angiogenic signaling through VEGFR deletion or VEGFR phosphorylation inhibition, which impairs venous thrombus resolution [23]. The observation that the degree of neovascularization of the fibrothrombotic material obstructing large pulmonary arteries is associated with the outcome in patients with CTEPH and the fact that angiogenesis inhibition is the reason behind misguided thrombus resolution suggests a role for deficient angiogenesis in the progression of this disease [22,94,95]. A study showed that the deletion of fetal liver kinase (Flk)-1 inhibits thrombus angiogenesis and delays thrombus resolution in mouse models of deep vein thrombosis [22]. Fibrinogen abnormalities may also play a role in thrombus development. Fibrinogen alteration increases thrombus development and slows thrombus decomposition, making the transition from thrombolysis to thrombus formation less efficient [96]. Fibrinogen genetic polymorphisms also have a role in unresolved blood clots. A study shows that the Thr312Ala (rs6050) polymorphism frequency in the fibrinogen alpha chain gene significantly differs between CTEPH and controls [97]. Other reports have shown mutations in the fibrinogen gene, including mutations in the β-chain P235L/γ-chain R375W, β-chain P235L /γ-chain Y114H, and β-chain P235L.

Additionally, mutations in the α-chain of the fibrinogen protein, specifically L69H and R554H, lead to a modified fibrin structure in clots that promotes thrombus non-resolution [18,19]. It is worth noting that patients with CTEPH had lower platelet counts, higher mean platelet volumes, and a greater tendency to platelet aggregation [98]. At the same time, the platelet endothelial cell adhesion molecule (PECAM)-1 deficiency has a role in CTEPH due to its involvement in leukocyte trafficking and inflammatory responses, which are crucial in thrombus resolution [21]. Patients with CTEPH also have chronic activation, consumption, and destruction of platelets. Notably, platelets of CTEPH patients have active GTP-binding GTPase RhoA compared to those in the non-CTEPH group, thus revealing a role for the P-selectin surface expression through PAC-1 binding that drives platelet aggregation [99].

In parallel, pulmonary arterial smooth muscle cells (PASMC) and endothelial cells (PAEC) from patients with CTEPH display altered features [100,101]. Besides disrupting the equilibrium between vasoconstriction and vasodilation, damage to the pulmonary endothelium can also start various events or processes that cause fast morphological and functional changes in the pulmonary vasculature [102]. The pulmonary endothelium is crucial to maintaining and preserving vascular function and integrity where the bloodstream meets the vessel wall. By secreting several mediators in response to chemical, mechanical, or physical stimuli, the pulmonary endothelium works as an active and dynamic receptor–effector tissue that improves gas exchange and barrier function, maintains the vasomotor and hemostatic balance, regulates the recruitment and retention of inflammatory cells, and ensures a functional angiogenic response [103,104].

The primary cause of the CTEPH onset is thrombus occlusion of the central pulmonary artery, leading to the occlusion of peripheral pulmonary arteries. Numerous pulmonary arteries become blocked after increasing blood flow to unblocked vessels, exerting stress pressure and injury on the artery wall. PH and vascular remodeling occur after these events [105]. The tunica media can constrict on its periphery, and the muscle arteries may transform into vein-like structures when a thrombus blocks the central pulmonary artery [106]. Tunica media disarrangement occurs through mechanical obstruction of the pulmonary arteries with organized fibrotic thrombi tightly adhered to the medial layer of the elastic pulmonary arteries that have replaced the normal intima [107]. We can note these lesions as a significant remodeling of the muscular pulmonary artery wall that measures around 50–500 µm, exhibiting a whole spectrum of PH-related lesions comparable to those noted in idiopathic pulmonary arterial hypertension, such as intimal fibromuscular proliferation and eccentric intimal fibrosis [79]. As in the case of a left-to-right cardiac shunt, when thrombi block proximal pulmonary arteries, pulmonary blood flow is redirected to unobstructed locations, creating localized high flow, pressure, and shear vascular stress [7]. Then, the thromboembolic component may obstruct the afflicted artery’s lumen, causing roughening of the intimal surface, the development of bands and webs that travel along the vascular lumen, and partial vascular recanalization [41]. The implications of this pulmonary artery obstruction include an increase in pulmonary vascular resistance, PH development, and eventually fatal right heart failure [108]. At first, evidence suggested that unresolved fibrotic clots causing intravascular blockage of pulmonary arteries were the exclusive cause of PH in patients with chronic progressive illness. Nonetheless, it is now widely acknowledged that pulmonary microvasculopathy, which contributes to the development of CTEPH, might result from pulmonary vascular remodeling [109]. These lesions may affect pulmonary capillaries and veins in addition to small muscular pulmonary arteries of less than 500 µm [8,110].

Microvasculopathy also develops due to anastomoses between the pulmonary and systemic circulation and hypertrophy and proliferation of the systemic bronchial arteries [109]. All small pre-capillary arteries, capillaries, and pulmonary venules exhibit CTEPH remodeling, like pulmonary hemangiomatosis and pulmonary veno-occlusive disease. These lesions are a distinctive feature of lung regions with distal to proximal pulmonary arteries occurring when organized clots block them entirely or partially [109,110]. A recent investigation revealed that the increased expression of miR-382-3p contributes to vascular remodeling by inhibiting ATG7 in the context of CTEPH [111]. A significant barrier to the effective treatment of this disease is the absence of an evident molecular basis for the mechanisms causing the microvasculopathy characteristic of CTEPH [69].

The elastic pulmonary arteries face a characteristic transformation from thromboembolic material into organized fibrotic scar tissue with tight attachments to the medial layer replacing the normal intima, leading to several degrees of stenosis, webs, and bands that complete occlusion of the vessel lumen [112]. When proximal pulmonary arteries become obstructed in different areas, the blood flow is redirected to unobstructed regions, causing localized high flow, pressure, and shear stress, like a left-to-right cardiac shunt [7]. Development of anastomoses between hypertrophy systemic bronchial arteries and pulmonary circulation downstream to pulmonary stenosis and occlusion may be an essential pathophysiological cause behind this phenomenon.

Molecular markers and signaling pathways in CTEPH are an emerging research field that aims to understand the disease better and identify novel indicators of CTEPH development. The nitric oxide (NO)–soluble guanylate cyclase (sGC)–cyclic GMP (cGMP) pathway is crucial for vascular function. Dysfunction in this pathway can lead to significant vascular abnormalities. For instance, vascular endothelium-derived NO inhibits platelet aggregation and smooth muscle cell growth. NO activates sGC to produce cGMP, a second messenger that induces smooth muscle relaxation. In patients with CTEPH, plasma levels of asymmetric dimethylarginine, an inhibitor of NO synthase, are elevated [27].

Additionally, elevated endothelin-1 (ET-1) levels promote smooth muscle cell proliferation within chronic clots in CTEPH and small-vessel disease [28]. In CTEPH, we observed reduced expression of Forkhead box class O transcription factor 1 (FoxO1), promoting the proliferation of PAECs and resulting in vascular remodeling [113]. Patients with CTEPH show overexpression of chemokine CXC ligand 13 (CXCL13) in pulmonary vascular lesions with critical pathogenic implications [114].

Aberrant long non-coding RNA (lncRNA) and microRNA (miRNA) expressions are crucial in the development of CTEPH [115,116]. PLXNA4 stimulates VEGF production with the basic fibroblast growth factor (b-FGF) signaling pathway that promotes angiogenesis [117]. The induction of epithelial–mesenchymal transition (EMT) by TGF-beta has consequences for lung diseases associated with fibrosis [33]. Heterozygous germline mutations in bone morphogenetic protein type II receptor (BMPR2) result in the downregulation of Smad signaling in PASMCs, thus losing its antiproliferative effect [29,30,31,32]. Additionally, somatic mosaicism in ACVRL1, a type I receptor for TGF-beta 1 that interacts with BMPR2, has been observed in patients with severe pulmonary hypertension [34]. Elevated endoglin (ENG) levels enhance the response to various ligands, including TGF-beta, Activin A, and BMP2. Since these ligands have a role in cardiovascular system development during embryogenesis, ENG may also contribute to remodeling during CTEPH [35]. Missense mutations in SMAD9, an essential component of the SMAD signaling pathway, are also involved in vascular remodeling [36]. Similarly, missense mutations in caveolin-1 (CAV1) affect its membranal functions, leading to increased constitutive endothelial permeability and reduced vascular endothelial cadherin (VE-cadherin) and β-catenin levels accompanied by loss of endothelial barrier function [37,38]. The increased expression of KCNK3 channels, crucial for maintaining the resting membrane potential in human PASMCs, contributes to hypoxic pulmonary vasoconstriction due to their pH-sensitive nature [39].

**Figure 4:**
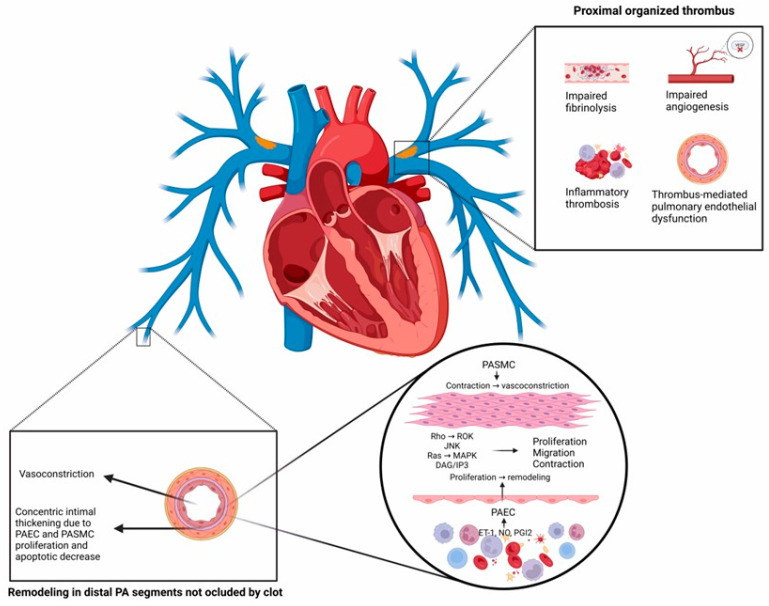
Summary of the main molecular mechanisms involved in developing chronic thromboembolic pulmonary hypertension. Central molecular mechanisms participate in CTEPH development, including the signaling pathways mediated by Rho, ROK, JNK, Ras, and MAPK. These molecular cascades lead to PASMC proliferation and migration into the intimal media, causing increased contraction and vasoconstriction in the pulmonary artery. ET-1, NO, and PIG2 activate PAEC proliferation and migration, contributing to concentric intimal thickening. Together with impaired fibrinolysis and angiogenesis, inflammatory thrombosis, and pulmonary endothelial dysfunction, increased vasoconstriction exacerbates vascular remodeling, generating proximal organized thrombus and remodeling of distal pulmonary artery segments not occluded by a clot. Finally, these changes lead to persistent pulmonary artery obstruction and increased pulmonary vascular resistance, two pivotal features of CTEPH. CTEPH: chronic thromboembolic pulmonary hypertension; PASMCs: pulmonary artery smooth muscle cells; Rho: GTP-binding GTPase RhoA; ROK: Rho-associated protein kinase; JNK: c-Jun N-terminal kinase; Ras: Ras GTPase superfamily; MAPK: mitogen-activated protein kinase; DAG: diacylglycerol; IP3: inositol phosphate; PAEC: pulmonary artery endothelial cells; ET-1: endothelin-1; NO: nitric oxide; PGI2: prostaglandin E2.

Thus, PLXNA4 and PMP22 may influence vascular growth and reconstruction, leading to CTEPH development [17]. Patients with CTEPH exhibit an upregulation of sodium voltage-gated channel alpha subunit 3 (SCN3A). The primary α-subunit that forms the functional Na+ channels in PASMC is SCN3A, which is extensively expressed in these cells [17]. Transmembrane ion circulation in the electrical excitability of PASMC cultured on control conditions is also a crucial component of excitation–contraction coupling in the pulmonary vascular system [118]. We outline the role of this molecular network in CTEPH development in Figure 4. A better understanding of these molecular pathophysiological mechanisms may help design novel biomarkers and clinical scales to identify patients at higher risk of developing CTEPH, even in the absence of PE or deep vein thrombosis.

## 7. Concluding Remarks

Recent advances in understanding the pathophysiology of CTEPH have led to a better understanding of the disease, which includes both proximal and distal obstruction of pulmonary arteries associated with the remodeling of muscular pulmonary arteries. This understanding goes beyond the pulmonary vascular disease caused by an intravascular mechanical obstruction due to chronic organized fibrotic material from unresolved clots. We now recognize several risk factors for CTEPH, but the mechanisms by which residual organized clots continue to form following an acute pulmonary embolism remain unclear. It is not yet entirely clear what causes fibrotic structured clots to be the only factor in the development of microvasculopathy. Developing more sensitive and specific diagnostic tests for CTEPH is essential to reduce the current diagnostic delay. Identifying specific biomarkers or imaging techniques for CTEPH could revolutionize early detection, including genetic prognostic factors. Furthermore, exploring additional risk factors for CTEPH, particularly in patients without a history of acute pulmonary embolism, is crucial to improving early detection and preventive clinical measures. Conducting long-term studies is also vital to comprehensively understanding the natural progression of CTEPH and its impact on patient quality of life.

## Figures and Tables

**Table 1:** Summary of the molecular mechanisms associated with chronic thromboembolic pulmonary hypertension development.

Mediator	Mechanism Involved	Possible Effect	Reference
*Fibrinogen and fibrinolytic abnormalities*			
Fibrinogen	Mutation in Aα-Thr312Ala of the fibrinogen protein sequence. Mutation in β-chain P235L/γ R375W of the fibrinogen protein sequence. Mutation in β-chain P235L/γ Y114H and P235L of the fibrinogen protein sequence. Mutation in α-chain L69H and R554H of the fibrinogen protein sequence.	These mutations lead to a modified fibrin structure in clots, promoting thrombus non-resolution. Patients with chronic thromboembolic pulmonary hypertension (CTEPH) share several fibrin abnormalities, where fibrin resists physiological thrombolysis, impairing thrombus resolution.	[18,19]
Plasminogen activator inhibitor-1 (PAI-1)	Increase in circulating levels	PAI-1 contributes to impaired clot dissolution, a key feature of the chronic nature of thromboembolism in CTEPH.	[20]
*Platelet function*			
Platelet endothelial cell adhesion molecule-1 (PECAM-1)	Deficiency	PECAM-1 is a glycopeptide receptor expressed in platelets, endothelial cells, and many other cell types. PECAM-1 is involved in leukocyte trafficking and inflammatory responses involved in thrombus resolution.	[21]
*Impaired angiogenesis*			
Fetal liver kinase-1 (Flk-1)	Deletion	Flk-1 deletion inhibits thrombus angiogenesis and delays thrombus resolution in mouse models of deep vein thrombosis.	[22]
*Endothelial function*			
Vascular endothelial growth factor (VEGF)	Receptor deletion	Experimental disruption of the endothelial angiogenic signaling via VEGF receptor (VEGFR) deletion or VEGFR phosphorylation inhibition impairs venous thrombus resolution.	[23]
*Inflammatory response*			
Interferon gamma-induced protein-10 (IP-10)	Increased expression	IP-10 increased expression promotes T cell adhesion to endothelial cells and inhibits bone marrow colony formation and angiogenesis.	[24]
Nuclear factor-kappa B (NF-κB)	Upregulation	NF-κB upregulation induces endothelial cell dysfunction in CTEPH and prompts increased expression of interleukin (IL-) 8, IL-1 beta, c-c motif chemokine ligand 5 (CCL5), and monocyte chemoattractant protein-1 (MCP-1).	[25,26]
*Small-vessel disease*			
Nitric oxide-soluble guanylate cyclase-cyclic guanosine monophosphate (NO–sGC–cGMP) pathway	Dysfunction	Vascular endothelium-derived NO inhibits platelet aggregation and smooth muscle cell growth. NO activates sGC to synthesize cGMP, a second messenger with many actions including smooth muscle relaxation. Plasma levels of asymmetric dimethylarginine, a NO synthase inhibitor, are increased in patients with CTEPH.	[27]
Endothelin-1 (ET-1)	Elevation	ET-1 promotes smooth muscle cell proliferation within chronic clots in CTEPH and small-vessel disease.	[28]
Bone morphogenetic protein type II receptor (BMPR2)	Heterozygous germline mutation	BMPR2 promotes pulmonary artery endothelial cell (PAEC) survival, thus protecting the pulmonary arteries from damage. Mutations of BMPR2 result in downregulation Smad signaling in pulmonary artery smooth muscle cells (PASMCs), with resultant loss of the antiproliferative effect.	[29,30,31,32]
Transforming growth factor-beta 1 (TGF-beta 1)	Increased expression	TGF-beta 1 promotes extracellular matrix protein production and epithelial–mesenchymal transition (EMT).	[33]
Activin A Receptor Type 1 (ACVRL1)	Somatic mosaicism	ACVRL1 is one of the TGF-beta 1 type I receptors interacting with BMPR2.	[34]
Endoglin (ENG)	Increased expression	ENG promotes response to ligands such as TGF-beta 1, Activin A, and BMPR2. Since ENG expression during embryogenesis is linked to cardiovascular system development, it may play a role in vascular remodeling in CTEPH.	[35]
Suppressor of Mothers against Decapentaplegic 9 (SMAD9)	Missense mutations	The SMAD9 signal pathway is associated with vascular remodeling.	[36]
Caveolin-1 (CAV1)	Missense mutation	CAV1 is a membrane protein of caveolae abundant in the vascular endothelium and other cells of the lung. CAV1 loss increases constitutive endothelial permeability and reduces vascular endothelial-cadherin (VE-cadherin) and β-catenin levels. Furthermore, loss of the endothelial barrier function is a significant phenomenon of inflammation.	[37,38]
Potassium channel subfamily K member 3 (KCNK3)	Increased expression	KCNK3 channels are major contributors to the resting potential in human PASMCs. KCNK3 are pH-sensitive channels responsible for driving modulatory effects in hypoxic pulmonary vasoconstriction.	[39]

Abbreviations: PAI-1: plasminogen activator inhibitor-1; PECAM-1: platelet endothelial cell adhesion molecule-1; Flk-1: fetal liver kinase-1; VEGF: vascular endothelial growth factor; IP-10: interferon gamma-induced protein-10; NF-κB: nuclear factor-kappa B; IL-: interleukin; CCL5: c-c motif chemokine ligand 5; MCP-1: monocyte chemoattractant protein-1; NO: nitric oxide; sGC: soluble guanylate cyclase; cGMP: cyclic guanosine monophosphate; ET-1: endothelin-1; BMPR2: bone morphogenetic protein type II receptor; PAECs: pulmonary artery endothelial cells; TGF-beta 1: transforming growth factor-beta 1; EMT: epithelial–mesenchymal transition; ACVRL1: Activin A Receptor Type 1; ENG: endoglin; CAV1: caveolin-1; KCNK3: potassium channel subfamily K member 3.

## Data Availability

Data are available upon request.
